# CIVIC ENGAGEMENT AMONG OLDER MIGRANTS IN EUROPE: EXAMPLES FROM FOUR EUROPEAN COUNTRIES

**DOI:** 10.1093/geroni/igad104.1676

**Published:** 2023-12-21

**Authors:** Sandra Torres, Pernilla Ågård, Emilia Häkkinen, Bas Dikmans, Inma Peiro-Milian, Rodrigo Serrat

**Affiliations:** Uppsala University, Uppsala, Uppsala Lan, Sweden; Uppsala University, Dept. of Sociology, Uppsala, Uppsala Lan, Sweden; Åbo Academy University, Vasa, Pohjanmaa, Finland; Vrije Universiteit Brussels, Brussels, Brussels Hoofdstedelijk Gewest, Belgium; University of Barcelona, Barcelona, Catalonia, Spain; University of Barcelona, Barcelona, Catalonia, Spain

## Abstract

Scholarship on old age social exclusion has identified civic engagement as a research area that deserves empirical attention. Research on older people’s civic engagement has in turn pointed out that experiencing life-course deficits in social capital, and/ or having had a life-course that does not resemble the continuity that social gerontology takes for granted, could put people at risk of becoming civically excluded in old age. These are two of the starting points for the European project known as CIVEX, which aims to study the life-course trajectories of civic engagement in groups of older people that have not yet received enough empirical attention. One of these groups is constituted by those who have migrated to the countries in which they are now based as adults, and whose life-course may therefore be more characterized by discontinuity rather than continuity. Departing from 60 life-course qualitative interviews with older migrants that are based in Sweden, Belgium, Spain and Finland, this presentation will draw attention to the ways in which these older people define civic engagement and the manner in which they describe how their migratory life-courses have impacted their civic engagement. The presentation will argue that there is theorizing potential embedded on the migratory life course, and that this could expand the scholarly imagination on old age social exclusion in general, and civic engagement in particular.

