# Correction to: CIVIC ENGAGEMENT AMONG OLDER MIGRANTS IN EUROPE: EXAMPLES FROM FOUR EUROPEAN COUNTRIES

**DOI:** 10.1093/geroni/igae008

**Published:** 2024-02-13

This is a correction to: Sandra Torres, Pernilla Ågård, Emilia Häkkinen, Bas Dikmans, Inma Peiro-Milian, Rodrigo Serrat, CIVIC ENGAGEMENT AMONG OLDER MIGRANTS IN EUROPE: EXAMPLES FROM FOUR EUROPEAN COUNTRIES, *Innovation in Aging*, Volume 7, Issue Supplement_1, December 2023, Page 510, https://doi.org/10.1093/geroni/igad104.1676

In the originally published version of this manuscript there was a typographical error in the fifth author’s name. This should read: “Inma Peiro-Milian”.

The emendation has been made in the article.

